# The complete mitogenome of the rockweed *Fucus distichus* (Fucaceae, Phaeophyceae)

**DOI:** 10.1080/23802359.2017.1310604

**Published:** 2017-04-07

**Authors:** Jeffery R. Hughey, Paul W. Gabrielson

**Affiliations:** a Division of Mathematics, Science, and Engineering, Hartnell College, Salinas, CA, USA;; b Biology Department and Herbarium, University of North Carolina at Chapel Hill, Chapel Hill, NC, USA

**Keywords:** Alaska, British Columbia, *cox1*, *Fucus vesiculosus*, Oregon

## Abstract

The rockweed *F. distichus* is a one of the most common intertidal seaweeds in the northern hemisphere. The systematics of *F. distichus* however remains open to discussion. Here, we contribute to the bioinformatics and systematics of *F. distichus* by deciphering its complete mitogenome. The *F. distichus* mitogenome is 36,400 bp in length, contains 67 genes, and has a gene content, organization, and sequence that are similar to the generitype, *F. vesiculusus*. These data support the continued recognition of *F. distichus* as a polymorphic entity with a broad distribution and high degree of ecological diversity.

*Fucus* L. is one of the three original genera proposed by Linnaeus (1753) to accommodate macroscopic marine algae. Since then more than 1000 names have been assigned to the genus (Guiry & Guiry 2017), but nearly all have been reclassified. Based on genetic analyses, three or four species are recognized: *F. distichus* L., *F. serratus* L., *F. spiralis* L., and *F. vesiculosus* L. (Serrao et al. 1999; Coyer et al. 2006; Kucera & Saunders 2008; Coyer et al. 2011; Laughinghouse et al. 2015). This classification remains problematic however, as exemplified by *F. distichus*, which displays a high degree of morphological, ecological, geographical, and reproductive variation (Gardner 1922; Kucera & Saunders 2008; Coyer et al. 2011; Laughinghouse et al. 2015). Herein, we characterize the mitogenome of *F. distichus* to clarify its systematics.

*Fucus distichus* (Voucher- UC 2050487) was collected from Coos Bay, Oregon (43°21′30.3″, −124°18′32.2″) and its DNA isolated following Lindstrom et al. (2011). The 76 bp paired-end library construction and sequencing was performed by myGenomics, LLC (Alpharetta, GA,) yielding 22,040,820 reads. The mitogenome was assembled using the default *de novo* settings in CLC Genomics Workbench 9.5 (^®^2016 CLC bio, a QIAGEN Company, Waltham, MA) and annotated using blastx and NCBI ORF-finder. The mitogenome was aligned to other Phaeophyceae with MAFFT (Katoh & Standley 2013). The RaxML analysis was executed using complete mitogenome sequences at Trex-online (Boc & Makarenkov 2012) with the GTR + gamma model and 1000 fast bootstraps, then visualized with TreeDyn 198.3 at Phylogeny.fr (Dereeper et al. 2008).

The *F. distichus* mitogenome (GenBank KY678904) is 36,400 bp in length and contains *cob* and *tat*C, 3 rRNA, 3 open reading frames, 3 ATP synthase, 3 *cox*, 6 *rpl*, 10 NADH, 11 *rps*, and 26 tRNA genes (*trn*L occurs in triplicate and *trn*I, *trn*M, *trn*S, *trn*Y in duplicate). Gene content, organization, and length are nearly identical to *F. vesiculosus* (36,392 bp) (Oudot-Le Secq et al. 2006). The genetic distance between these two species is 2.0%. Phylogenetic analysis of *F. distichus* supports this close relationship, positioning it in a clade with *F. vesiculosus* (Figure 1). Sequence analysis of *F. distichus* from Oregon identifies it as haplotype *ic6* (Coyer et al. 2011) from Japan and Alaska, USA. Its *cox*1 sequence was also an exact match to a specimen from British Columbia, Canada (GenBank EU646633), and only differed by 1 bp from *F. distichus* from Nova Scotia, Canada (GenBank EU646647), Nordland, Norway (GenBank LN877838), and California, USA (GenBank KM254965).

**Figure 1.**
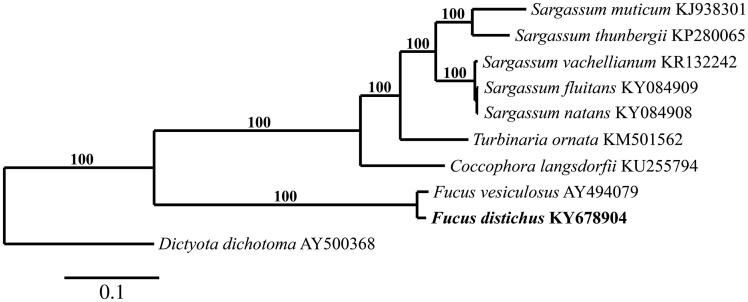
Maximum-likelihood phylogram of *F. distichus* (bold letters) and related Phaeophyceae mitogenomes. Numbers along branches are RaxML bootstrap supports based on 1000 nreps. The legend below represents the scale for nucleotide substitutions.
